# Epidemiological and serological surveillance of hand-foot-and-mouth disease in Shanghai, China, 2012–2016

**DOI:** 10.1038/s41426-017-0011-z

**Published:** 2018-01-24

**Authors:** Jiayu Wang, Zheng Teng, Xiaoqing Cui, Chongshan Li, Hao Pan, Yaxu Zheng, Shenghua Mao, Yuying Yang, Limeng Wu, Xiaokui Guo, Xi Zhang, Yongzhang Zhu

**Affiliations:** 1 Department of Microbiology and Immunology, Institutes of Medical Science, Shanghai Jiao Tong University School of Medicine, Shanghai, China; 2 Microbiology Laboratory, Shanghai Municipal Centre for Disease Control and Prevention, Shanghai, China; 3 Expanded Program on Immunization Laboratory, Shanghai Municipal Centre for Disease Control and Prevention, Shanghai, China; 4 Department of Clinical Microbiology, Institute of Antibiotics, Huashan Hospital, Fudan University, Shanghai, China

## Abstract

Aside from enterovirus 71 (EV71) and coxsackie virus A16 (CV-A16), viruses that are known to cause hand-foot-and-mouth disease (HFMD), epidemiological profiles of other enteroviruses that induce HFMD are limited. We collected 9949 laboratory surveillance HFMD cases and 1230 serum samples from infants and children in Shanghai from 2012–2016. Since 2013, CV-A6 has displaced EV71 and CV-A16 to become the predominant serotype. Interestingly, novel epidemiological patterns in EV71 and CV-A16 infections were observed, with one large peak in both 2012 and 2014, followed by two smaller peaks in the respective following years (2013 and 2015). Through sequencing, we found that C4a, B1b, D-Cluster-1 and B constituted the major subgenotypes of EV71, CV-A16, CV-A6 and CV-A10, respectively. Among healthy individuals, 50.49% and 54.23% had positive neutralising antibodies (NtAbs) against EV71 and CV-A16, respectively, indicating that EV71 and CV-A16 silent infections were common. These populations may be an important potential source of infection. The overall seropositive rate of EV71 NtAbs showed a fluctuating, markedly downward trend, indicating the potential risk of a future EV71 epidemic. High CV-A16 NtAb seroprevalence corroborated a documented CV-A16 ‘silent’ epidemic. Children aged 1–5 years had the lowest EV71 NtAb seropositive rate, whereas those aged 1–2 years exhibited the lowest CV-A16 NtAb seropositive rate. This is the first comprehensive investigation of the epidemiology and aetiology, as well as the seroprevalence, of HFMD in Shanghai between 2012 and 2016. This study provides the latest insights into developing a more efficient HMFD vaccination programme.

## Introduction

Hand-foot-and-mouth disease (HFMD) is a common infectious disease of global concern, caused by a wide spectrum of human enteroviruses (EV), particularly enterovirus 71 (EV71) and coxsackie virus A16 (CV-A16)^1^. HFMD infections generally manifest mild symptoms such as fever, erythrasma, vesiculation and inappetence, occasionally giving rise to severe symptoms, including central nervous system damage, encephalomyelitis, aseptic meningitis, acute flaccid paralysis and even death^2^. Children under 5 years old are the most susceptible to HFMD. As a C-class notifiable communicable disease, the Chinese national surveillance system for HFMD mainly focuses on EV71 and CV-A16 due to their predominance; however, little is known about the epidemiology of other human enteroviruses that cause HFMD, including CV-A6, A10, A2, A4, A5, A8, A12, A14 and CV-B1–B5^3–5^. Since 2012, outbreaks of other HEV-A strains, such as CV-A6 and CV-A10, have been frequently reported in Edinburgh, Finland, France and Japan^6–11^. In many cities in China, including Beijing, Tianjin, Jilin, Nanjing, Guangzhou, Shenzhen, Qingdao^12–18^, CV-A6 has been found to account for an increasing number of HEV-A outbreaks. In both severe and mild cases, EV71 was the predominant causative agent during the 2008–2011 survey periods, whereas other enteroviruses became predominant after 2011 in Shanghai, Jiangsu, Hubei, Hunan and Ningxia provinces^19^. Despite the large-scale CV-A6 outbreak associated with HFMD in China, the epidemiological characteristics and aetiological features in Shanghai and other cities remain unclear.

Shanghai is located in southeast China and is the country’s second largest city with 24 million permanent residents and 9 million migrants. Since 2009, the Shanghai Municipal Centre for Disease Control and Prevention (Shanghai CDC) has established its own epidemiological surveillance system. Sixteen local CDC representing 16 districts are responsible for collecting samples and transporting them in their own districts. Per Shanghai’s HFMD monitoring programme, clinical specimens from at least five outpatients are diagnosed with HFMD per month at local sentinel hospitals in each district. Throat swabs or/and faecal samples (rectal swabs) are then sent directly to microbiology labs at the local CDCs, where HEV, EV71 and CV-A16 can be confirmed by real-time PCR. Since 2013, CV-A6 and CV-A10 detection has been added to further identify HEV serotypes. Finally, all specimens and related case information are then sent to the Shanghai CDC for subsequent virus isolation and routine sequencing analysis.

In this study, we collected 9949 laboratory surveillance HFMD cases and 1230 serum samples from healthy individuals in Shanghai between 2012 and 2016 to determine the current epidemiological features of HFMD in Shanghai and to investigate the antibodies against EVs associated with HFMD. Moreover, since the EV71 vaccine was introduced in Shanghai last year, our study contributes to developing effective prevention and control measures and provides the latest insights into establishing an immunisation programme against HFMD in Shanghai.

## Materials and methods

### Ethics statement

The protocols in this study were screened and approved by the ethics committees of the Shanghai CDC. Clinical specimens, including throat swabs, faeces and blood, were obtained from the routine HFMD surveillance programme of the Shanghai CDC. Sample collection was approved by either the patients or their parents with prior informed consent.

### Case definition

The criteria for clinical HFMD diagnosis were established per the official National Guidelines for the Diagnosis and Treatment of HFMD in China (2009 edition): (http://www.chinacdc.cn/jkzt/crb/szkb/jszl_2275/200906/t20090612_24707.htm). Mild cases were defined as those having oral ulcers and maculopapular or vesicular rashes on the hands, feet and buttocks, with or without fever. Severe cases were defined as those with additional symptoms including neurological and/or cardiopulmonary complications such as encephalitis, aseptic meningitis, encephalomyelitis, myoclonus, acute flaccid failure, pulmonary oedema, pulmonary haemorrhage or cardiorespiratory failure. Healthy individuals were defined as those who had no oral ulcers, maculopapular or vesicular rashes on the hands, feet or buttocks, fever, cough or other respiratory syndromes with no history of HFMD infection.

### RNA extraction and virus identification

Throat swabs and faeces were used for RNA extraction, enterovirus detection and virus isolation. Throat swabs were preserved in a solution containing 5% FBS. MEMs were used to dilute faecal samples into 10% suspensions. After mixing thoroughly, 200 μl clinical samples were measured for RNA extraction using the Roche MagNA Pure LC 2.0 nucleic extraction system (ROCHE, CO, USA), per the manufacturer’s instructions. Pan EV, EV71, CV-A16, CV-A6 and CV-A10 were confirmed using a commercial real-time RT-PCR Kit (BioPerfectus technologies, Jiangsu, China).

### Virus isolation and sequencing

Strains were isolated from EV RNA-positive samples from a human rhabdomyosarcoma (RD) cell line and cultured at 37 °C for 5–7 days. The culture lysate was harvested and passaged into a fresh monolayer of RD cells and observed for another 5–7 days. When cytopathic effects (CPE) occurred, the culture medium and cells were collected for purification.

For VP1 sequencing, PCR was performed using an Invitrogen Onestep RT-PCR kit and specific primers^16,20–23^ (Table S1). In detail, for EV71, CV-A16 and CV-A6 isolates, full-length VP1 genes were amplified using specific primers. For CV-A6 clinical specimens, partial VP1 genes were amplified using pan EV-VP1 primers. Partial CVA-10 VP1 genes were amplified using 486 and 488 primers.

### Phylogenetic analysis

Phylogenetic trees were constructed using the neighbour-joining method in MEGA version 6.0 software^24^. Sequences were aligned using Clustal W with the Kimura-2 parameter. Tree robustness was determined by bootstrapping using 1000 pseudo replicates.

### Neutralising antibody detection

A total of 1230 serum samples from healthy individuals (698 males and 532 females) were divided into four different age groups (0–1, 1–2, 3–5, 6–18) and used to detect neutralising antibody titres (NtAb) against EV71 and CV-A16 using a micro-neutralisation test. Serum samples were collected in four local districts (Changning, Yangpu, Qingpu, Minhang) in Shanghai between 2014 and 2016, before and after the HFMD epidemic periods. The pre-HFMD epidemic period was defined as March to May, and the immediate post-HFMD epidemic period was defined as September to November.

Neutralising antibodies against EV71 and CV-A16 were detected in a human RD cell line per the official Guidelines of the Diagnosis and Treatment of HFMD (2009 edition) with some modifications. To define the EV71-neutralising antibody, the EV71 clinical strain (evolutionary branch: C4a; GenBank accession number: EU703812) was isolated from one HFMD patient in Anhui province in 2008. The CV-A16 clinical strain (evolutionary branch: B1b; GenBank accession number: GQ429229) was isolated from one Shandong HFMD patient in 2007^21^. Serum samples were inactivated at 56 °C for 30 min before use, and samples were diluted at 1:8, 1:16, 1:64, 1:256 and 1:1024. Fifty microliters of the virus, with a tissue serial culture infective dose (TCID50) of 100, was mixed with 50 μl of the appropriate serum dilution and incubated at 37 °C for 2 h. After incubation, 1 × 10^5^ RD cells per ml were added to each well. Finally, the plates were incubated in a 5% CO_2_ incubator at 37 °C for 7 days. CPE was observed under an inverted microscope starting on the fourth day. All diluted samples were tested in duplicate. Cell and virus controls were included in each plate. Viral back titration was conducted with each test. An antibody serum titre of more than 8 was considered positive, and the geometric mean titre (GMT) was also calculated.

### Statistical analysis

All statistical analyses were performed using R software (https://www.r-project.org/). All statistical tests were two-sided, and a *P*-value < 0.05 was considered statistically significant. Neutralising antibody titres from positive serum samples were log-transformed to calculate the GMT and 95% confidence intervals (CI). A *χ*^2^ test was used to compare the distribution of EV71 and CV-A16 NtAb-positive rates for the groups categorised by age, gender and specimen collection time. All titres below 8 were assumed to be 4 for calculation. Titres ≥1024 were assigned the value of 1024.

## Results

### Epidemiological features of HFMD in Shanghai

**Fig. 1:**
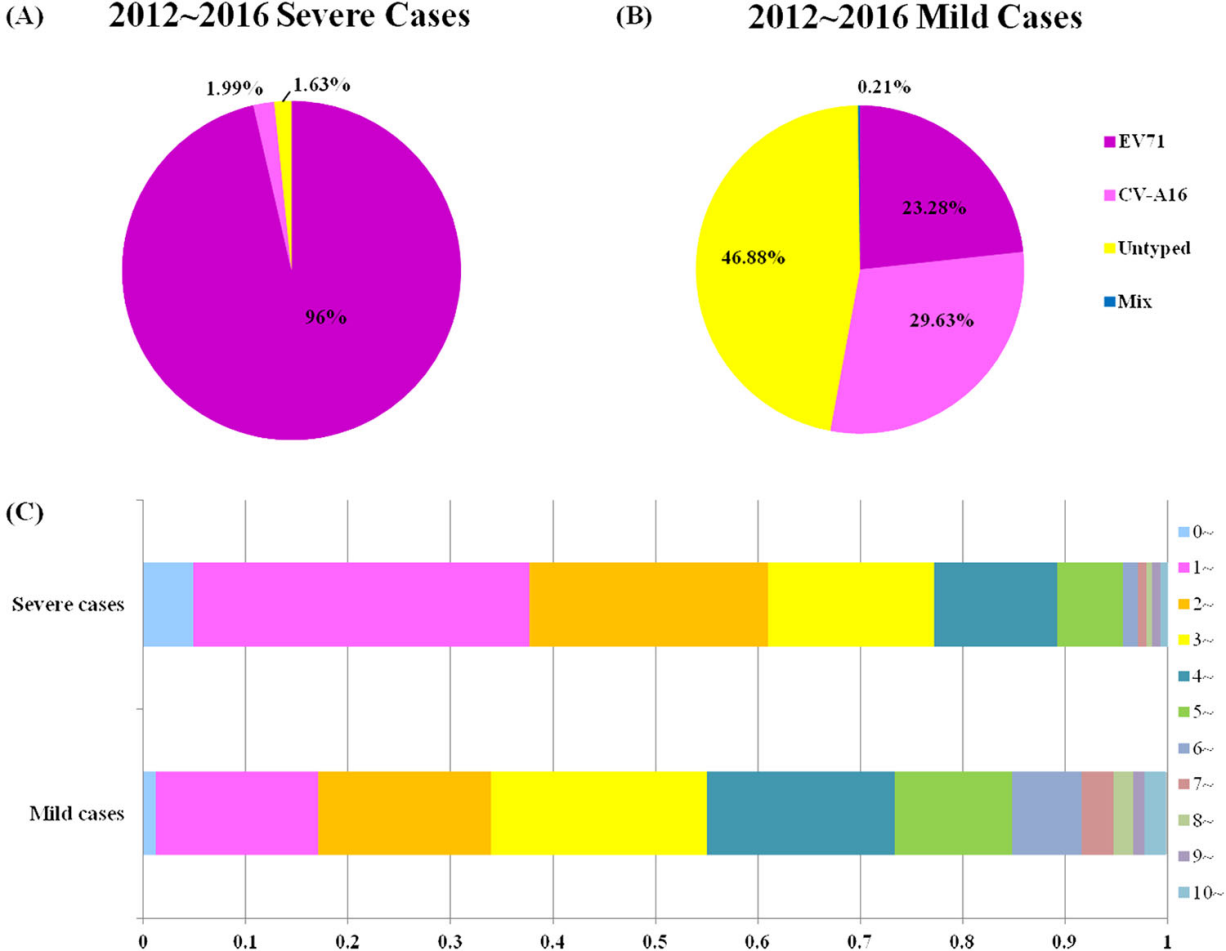
Percentages of EV71, CV-A16 and untyped enterovirus isolated from HFMD cases in 2012 to 2016 in Shanghai, China. **a** Severe cases. **b** Mild cases. **c** Age distribution of HFMD mild and severe cases from 2012 to 2016 in Shanghai. As CV-A6 and CV-A10 was not detected for further EV-serotype identification until 2013, the untyped EV for 2012–2016 shown here may include CV-A6 and CV-A10 infections

A total of 9949 laboratory surveillance HFMD cases in Shanghai from January 2012 to December 2016 were included in this study. Of these cases, 9357 (94.05%) presented mild symptoms, whereas 592 (5.95%) were diagnosed with severe HFMD with neurological or cardiopulmonary complications. EV71 was significantly associated with severe HFMD (533 of 553 (96.38%) EV-positive severe HFMD cases) compared to CV-A16 (11 of 553 (1.99%) EV-positive cases) (*P* < 0.05) (Fig. 1a). In addition, nine EV-positive HFMD cases were severe but were EV71, CV-A16, CV-A6 and CV-A10-negative. However, HFMD causative agent distribution in mild cases showed that EV-positive cases that were also EV71 and CV-A16-negative cases predominated from 2012 to 2016, comprising 46.88% of the mild cases (3400 of 7252 EV-positive cases) (Fig. 1b). The annual proportions of EV-positive but EV71 and CV-A16-negative cases were 21.90%, 66.04%, 29.56%, 55.56% and 51.87% for 2012–2016, respectively, with significant fluctuations over the past 5 years (Table 1). For convenience, we defined EV-positive but EV71 and CV-A16 negative cases as ‘untyped EV infections’. Owing to the increase in untyped EV case reports during the last half of 2012, CV-A6 and CV-A10 detection was added in 2013 to further identify EV serotypes in the routine HFMD laboratory surveillance programme. Since then, untyped EV cases represent EV-positive but EV71, CV-A16, CV-A6 and CV-A10-negative cases as untyped EV infections. Thus, the untyped EV in 2012 may include some CV-A6 and CV-A10 infections. The gender distribution ratio for male to female cases was ~1.54 and reached 1.96 for severe cases. In males, 62.94% (1398 of 2221), 59.17% (1278 of 2160) and 63.24% (2156 of 3409) were caused by EV71, CV-A16 and untyped EV, respectively.

**Table 1:** Pathogen distribution of HFMD cases in Shanghai, China, 2012–2016

Year	2012	2013	2014	2015	2016
Case number	2131	1780	2272	1610	2156
Severe cases (%)	174 (8.17)	50 (2.81)	217 (9.55)	46 (2.86)	105 (4.87)
Male (%)	1278 (59.97)	1076 (60.45)	1392 (61.27)	977 (60.68)	1309 (60.71)
Age (median)	4	4	3	3	3
pan EV-positive	1571	1328	1810	1276	1820
EV71 (%)	589 (37.49)	321 (24.17)	621 (34.31)	274 (21.47)	416 (22.86)
CV-A16 (%)	638 (40.61)	128 (9.64)	644 (35.58)	290 (22.73)	460 (25.27)
Untyped (%)	344 (21.90)	236 (17.77)	128 (7.07)	66 (5.18)	84 (4.62)
CV-A6 (%)	NA^a^	584 (43.98)	390 (21.55)	605 (47.41)	831 (45.66)
CV-A10 (%)	NA^a^	59 (4.44)	27 (1.49)	41 (3.21)	29 (1.59)

^a^In 2012, detection of CV-A6 and CV-A10 was not implemented. The untyped EV in 2012 was defined as pan EV-positive but EV71 and CV-A16 negative cases. Since 2013, the untyped EV was defined as pan EV-positive but EV71, CV-A16, CV-A6 and CV-A10 negative cases.

The average patient age was 3.55 years, and the median age of onset was 3 years. The average ages for EV71, CV-A16 and untyped EV infections were 3.38, 3.70 and 3.44 years, respectively. Age distribution showed that children under 5 years old accounted for more than 84.81% of mild cases and 95.61% of severe cases. In summary, 3- to 4-year-old accounted for 55% of mild cases and 77.20% of severe cases; 1- to 2-year-old accounted for 33.95% of mild cases and 60.98% of severe cases; and children over 5 years old accounted for 15.07% of mild cases and 4.39% severe cases. 3- to 4-year-old represented the highest incidence in mild cases, while 1 to 2-year-olds comprised the highest incidence of severe cases (Fig. 1c).

### Novel epidemic patterns in HFMD aetiology

**Fig. 2:**
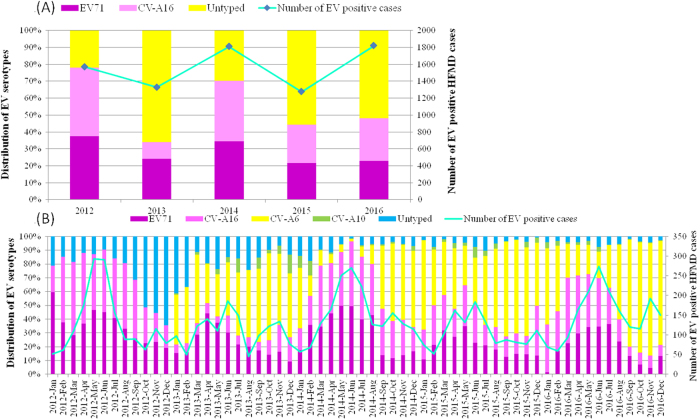
Enterovirus-associated HFMD cases and enterovirus distribution in Shanghai, China from 2012 to 2016. **a** Continuous lines represent the number of EV-positive HFMD cases; the histogram shows the percentages of EV71, CV-A16 and untyped enterovirus isolated from EV-positive samples. **b** Monthly distribution of enterovirus serotypes from HFMD cases in Shanghai from 2012 to 2016. *In **a**, all the untyped EV refers to pan EV-positive but EV71- and CV-A16-negative samples. In **b**, the untyped EV samples in 2012 correspond to pan EV-positive but EV71- and CV-A16-negative samples, and untyped EV samples from 2013 to 2016 correspond to pan EV-positive but EV71, CV-A16, CV-A6 and CV-A10-negative samples

Per our laboratory surveillance system, EV71 and CV-A16 represented the two major circulating enterovirus serotypes in Shanghai between 2007 and 2011, comprising ~20.07% of all EV-positive infections^25,26^. EV71 and CV-A16 remained the leading serotypes in 2012; however, untyped EV replaced EV71 and CV-A16 as the major serotype in 2013, accounting for over 50% of EV-positive HFMD cases (Fig. 2a).

In addition to CV-A6 becoming the predominant EV-serotype associated with HFMD, changes have occurred in the EV71 and CV-A16 epidemiology. Regular fluctuations in EV71 and CV-A16 infection proportions were observed, with one large peak in one year and two smaller peaks in the following year. One large peak each occurred in 2012 and 2014, lasting from April to August. The EV71 and CV-A16 infection peak proportion comprised over 80% of the EV-positive HFMD cases. However, in 2013 and 2015, there were two small peaks; the larger peak lasted from March to June and two smaller peaks occurred in the winter. The large peak, whose proportion reached 50%, reflected the summer HFMD epidemic (Fig. 2b).

Interestingly, a higher incidence of severe HFMD cases also occurred when EV71 and CV-A16 infections formed one large peak. The incidences of severe cases (percent of all cases) were 8.17% and 9.55% in 2012 and 2014 with only one peak, whereas in 2013 and 2015, two small peaks occurred in only 2.81% and 2.86% of severe HFMD cases (*P* < 0.05) (Table 1). In addition, during the large peak caused by EV71 and CV-A16, the untyped EVs, particularly CV-A6, accounted for only 10% of HFMD cases in the spring and summer of each year, whereas the CV-A6-positive rate was more than 50% during the following years with two small peaks.

The severe HFMD case incidence was related to variation in pathogen subtype frequencies and the EV-serotype distribution throughout the year. In 2016, a large complex peak of EV71 and CV-A16 infections occurred from March to July and both were responsible for ~70% of the EV-positive infections during the peak period. However, the incidence of severe cases was 4.87%, which was higher than the incidences in 2013 and 2015, but lower than those in 2012 and 2014 (*P* < 0.05). This may be due to the different EV-serotype distributions throughout the year. In 2012 and 2014, EV71 and CV-A16 became the major serotypes, whereas in 2013 and 2015, untyped EV (mostly CV-A6) became the predominant causative agent of HFMD (Fig. 2a).

Since 2013, untyped EVs, especially CV-A6, have become the predominant HFMD agents in autumn and winter, accounting for over 60% of these cases every 2 years, whereas EV71 or CV-A16 infections are most prevalent in warm seasons, indicating that higher temperatures are vital for these two serotypes.

### VP1 sequencing and phylogenetic analysis

211 EV71, 232 CV-A16, 66 CV-A6 (including 12 total VP1 sequences of 915 bps and 54 partial VP1 sequences of 324 bps) and 12 CV-A10 strains (with partial VP1 sequences of 572 bps) were isolated, and their VP1 genes were sequenced. As the multiplication of CV-A6 in RD cell lines was unsatisfactory (data not shown), a partial VP1 sequence from CV-A6-positive clinical specimens was amplified using CODEHOP primers^23^. All VP1 sequences were submitted to GenBank (accession nos. KX871238-KX871689 and KY972267-KY972320).

**Fig. 3:**
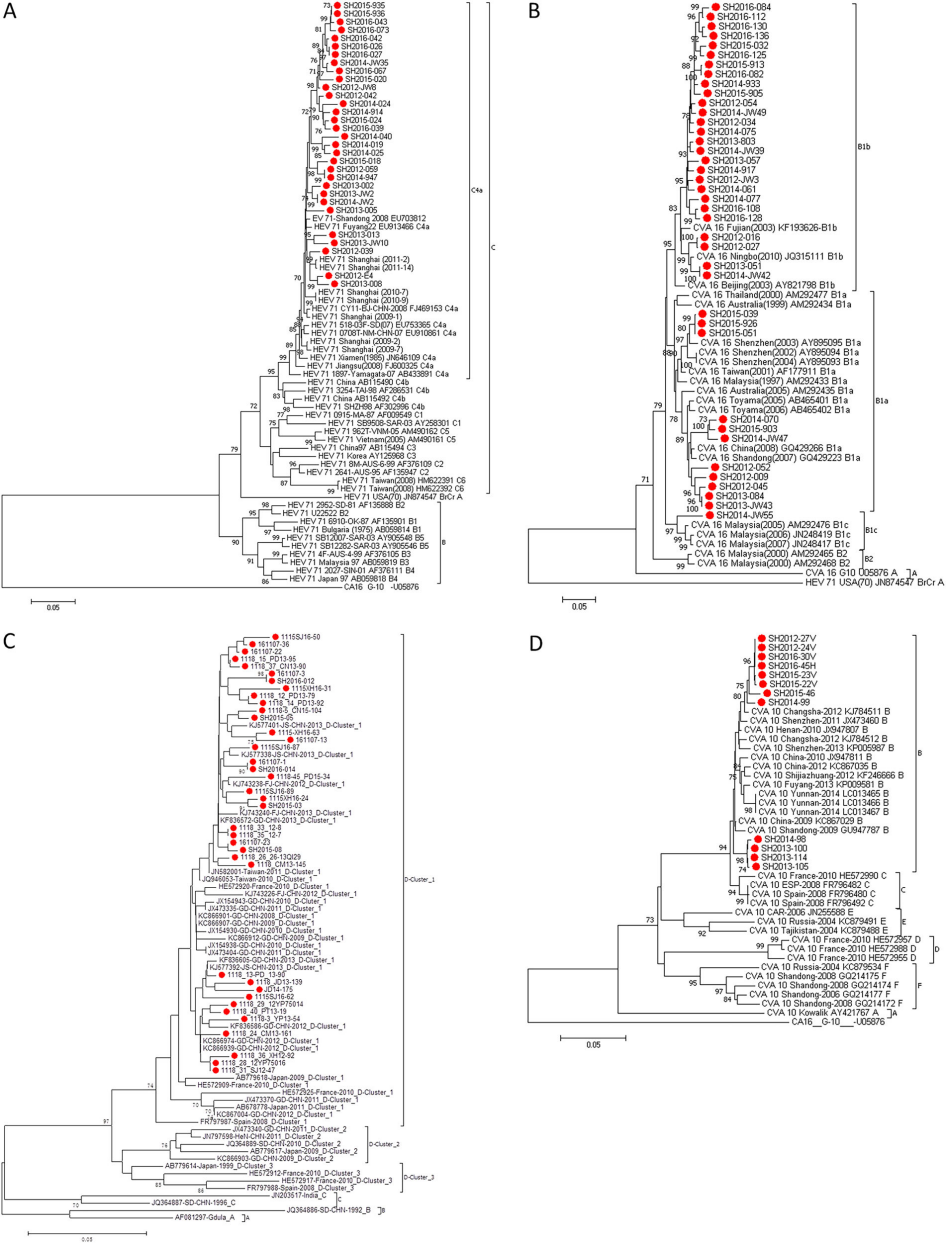
**Phylogenetic analysis of EV71, CVA16, CV-A6 and CV-A10 circulating in Shanghai in 2012-2016. a** Total VP1 gene sequences from 31 EV71 strains (891 bps) in this study and 41 reference sequences downloaded from GenBank were used for phylogenetic analyses. EV71 strains circulating in Shanghai in 2012–2016 belonged to the C4a cluster. **b** Total VP1 gene sequences from 39 CV-A16 strains (891 bps) in this study and 22 reference sequences downloaded from GenBank were used for phylogenetic analyses. CV-A16 strains circulating in Shanghai in 2012–2016 belonged to B1a and B1b clusters. **c** Partial VP1 gene sequences from 38 CV-A6 strains (354 bps) in this study and 42 reference sequences downloaded from GenBank were used for phylogenetic analyses. CV-A6 strains circulating in Shanghai in 2012–2016 belonged to D-cluster 1. **d** Partial VP1 gene sequences from 12 CV-A10 strains (572 bps) in this study and 31 reference sequences downloaded from GenBank were used for phylogenetic analyses. CV-A10 strains circulating in Shanghai in 2012–2016 belonged to B cluster.

All VP1 sequences (891 bps) from 41 EV71 strains and 55 CV-A16 strains in this study were used for phylogenetic analysis. The results revealed that both EV71 and CV-A16 circulated continuously from 2012–2016. Genotyping revealed that the endemic circulation pattern was punctuated by two groups: subgenogroup C4a for EV71 and B1a-B1b for CV-A16 (Fig. 3a, b). All EV71 isolates in this study belonged to evolutionary branch C4a (Fig. 3a). The CV-A16 isolates from Shanghai belonged to evolutionary branches B1a and B1b (Fig. 3b), whereas B1b of CV-A16 was the predominant genotype circulating in Shanghai. Only one B1c strain from CV-A16 was isolated by monitoring specimens in the Changning District of Shanghai in 2014, which was closely related to strains identified in Malaysia.

65 CV-A6 strains (324 bps) and 12 CV-A10 strains (572 bps) were used for phylogenetic analysis. The results showed that all CV-A6 strains in this study that were located in the D cluster 1 genotype were closely related to strains found previously in Guangdong, Fujian and Jiangsu (Fig. 3c). The CV-A10 strains from the B genotype were mainly distributed among two branches, eight of which were closely related to strains detected in Changsha in 2012, and four were closely related to strains detected in Shandong in 2009 (Fig. 3d).

### Time-dependent seroprevalence and titre distribution of NtAb against EV71 and CV-A16

Serum samples in this study were collected from March to May (before the HFMD epidemics) and September to November (after the HFMD epidemics) in 2014–2016. Demographic profiles of the study participants are provided in Table 2.

**Table 2:** Demographic profile of the subjects in three years studied

	2014 before epidemics (Mar. to May)	2014 after epidemics (Sep. to Nov.)	2015 before epidemics (Mar. to May)	2015 after epidemics (Sep. to Nov.)	2016 before epidemics (Mar. to May)	2016 after epidemics (Sep. to Nov.)
	No. samples	No. samples	No. samples	No. samples	No. samples	No. samples
Age groups (years)						
0–1	52	30	38	37	40	29
1–2	101	60	41	43	40	29
3–5	127	50	41	40	80	32
6–18	20	30	80	80	80	60
Gender						
Male	171	116	106	111	118	76
Female	129	64	94	89	82	74
Total	300	180	200	200	200	150

The EV71 and CV-A16 infection rates were relatively high in the studied population, although all serum samples were collected from healthy individuals who stated that they were never infected with HFMD. During the past three years, 50.49 and 54.23% of these healthy individuals had, respectively, been exposed to EV71 or CV-A16. Of these, 35.61% had both EV71 and CV-A16-positive NtAbs, indicating that over one-third of them had been exposed to one of the two viruses (Table 3).

**Table 3:** Overall seroprevalence of HEV 71 and/or CVA 16 antibody

Year	No. of tested	No. (%) of positive		
		HEV 71	CVA 16	Co-infection
2014 (before)				
Male	171	80 (46.78)	93 (54.39)	58 (33.92)
Female	129	71 (55.04)	75 (58.14)	54 (41.86)
Total	300	151 (50.33)	168 (56.00)	112 (37.33)
2014 (after)				
Male	116	83 (71.55)	71 (61.21)	58 (50.00)
Female	64	48 (75.00)	40 (62.50)	35 (51.56)
Total	180	131 (72.78)	111 (61.67)	93 (50.56)
2015 (before)				
Male	106	46 (43.40)	60 (56.60)	35 (33.02)
Female	94	41 (43.62)	42 (44.68)	27 (28.72)
Total	200	87 (43.50)	102 (51.00)	62 (31.00)
2015 (after)				
Male	111	76 (68.47)	87 (78.38)	61 (54.95)
Female	89	53 (59.55)	63 (70.79)	42 (47.19)
Total	200	129 (64.50)	150 (75.00)	103 (51.50)
2016 (before)				
Male	118	41 (34.75)	29 (24.58)	9 (7.63)
Female	82	23 (28.05)	21 (25.61)	11 (13.41)
Total	200	64 (32.00)	50 (25.00)	20 (10.00)
2016 (after)				
Male	76	27 (35.53)	46 (60.53)	21 (27.63)
Female	74	32 (43.24)	40 (54.05)	27 (36.49)
Total	150	59 (39.33)	86 (57.33)	48 (32.00)
Overall				
Male	698	353 (50.57)	386 (55.30)	242 (34.67)
Female	532	268 (50.38)	281 (52.82)	196 (36.84)
Total	1230	621 (50.49)	667 (54.23)	438 (35.61)

**Fig. 4:**
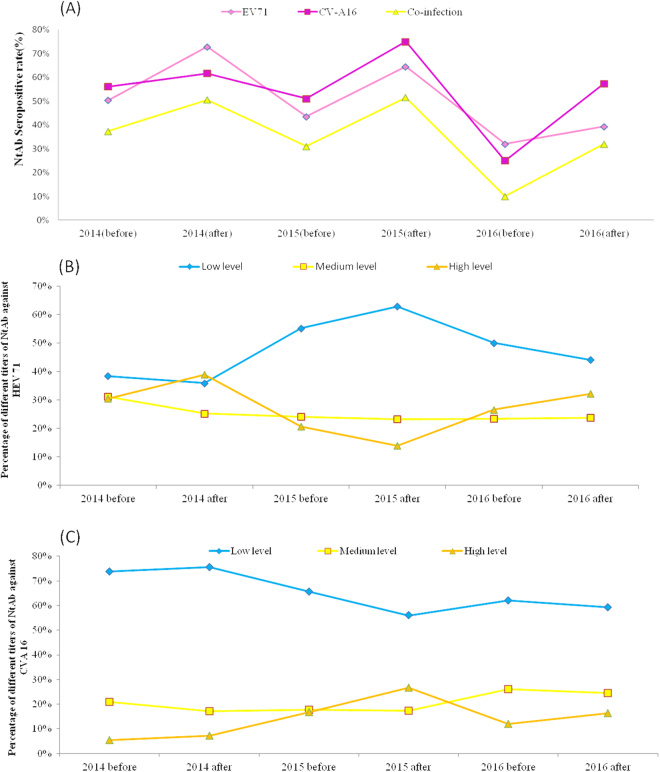
**Seropositive rate of neutralizing antibody against EV71 and CV-A16 in healthy individuals. a** Overall neutralising antibody seropositive rate in healthy individuals in Shanghai during 2014–2016. **b**–**c** EV71 and CV-A16 neutralising antibody levels from healthy individuals in Shanghai during 2014–2016 before and after the HFMD epidemics.

To analyse antibody titres, three NtAb titre ranges were defined: 8–32 (low), 64–256 (medium) and 512–1024 (high)^27^. Our results showed that the serum specimens collected after the HFMD epidemics had higher seropositive rates for EV71 and CV-A16 NtAb than those from samples collected during the pre-epidemic periods (Fig. 4a). During 2014–2016, the overall EV71 seropositive rate showed a fluctuating downward trend, indicating a high risk for future EV71 infection. The percentage of high-level NtAb titres against EV71 peaked in the post-epidemic periods in 2014 and 2016, suggesting large-scale asymptomatic infections in healthy individuals during the 2014 and 2016 HFMD epidemics (Fig. 4b). Compared to EV71, the highest CV-A16 NtAb seroprevalence occurred during the post-HFMD epidemic period in 2015. The percentage of high-level NtAb titres against CV-A16 peaked in the same period (Fig. 4c), corroborating a documented CV-A16 epidemic. A high seropositive CV-A16 NtAb infection rate also occurred after the 2016 HFMD epidemic (Fig. 4a), whereas the percentage of medium and high-level titres showed an uptrend in the same period, which may have resulted from a recent infection in this group.

### Age-dependent immunity to EV71 and CV-A16 infections

**Fig. 5:**
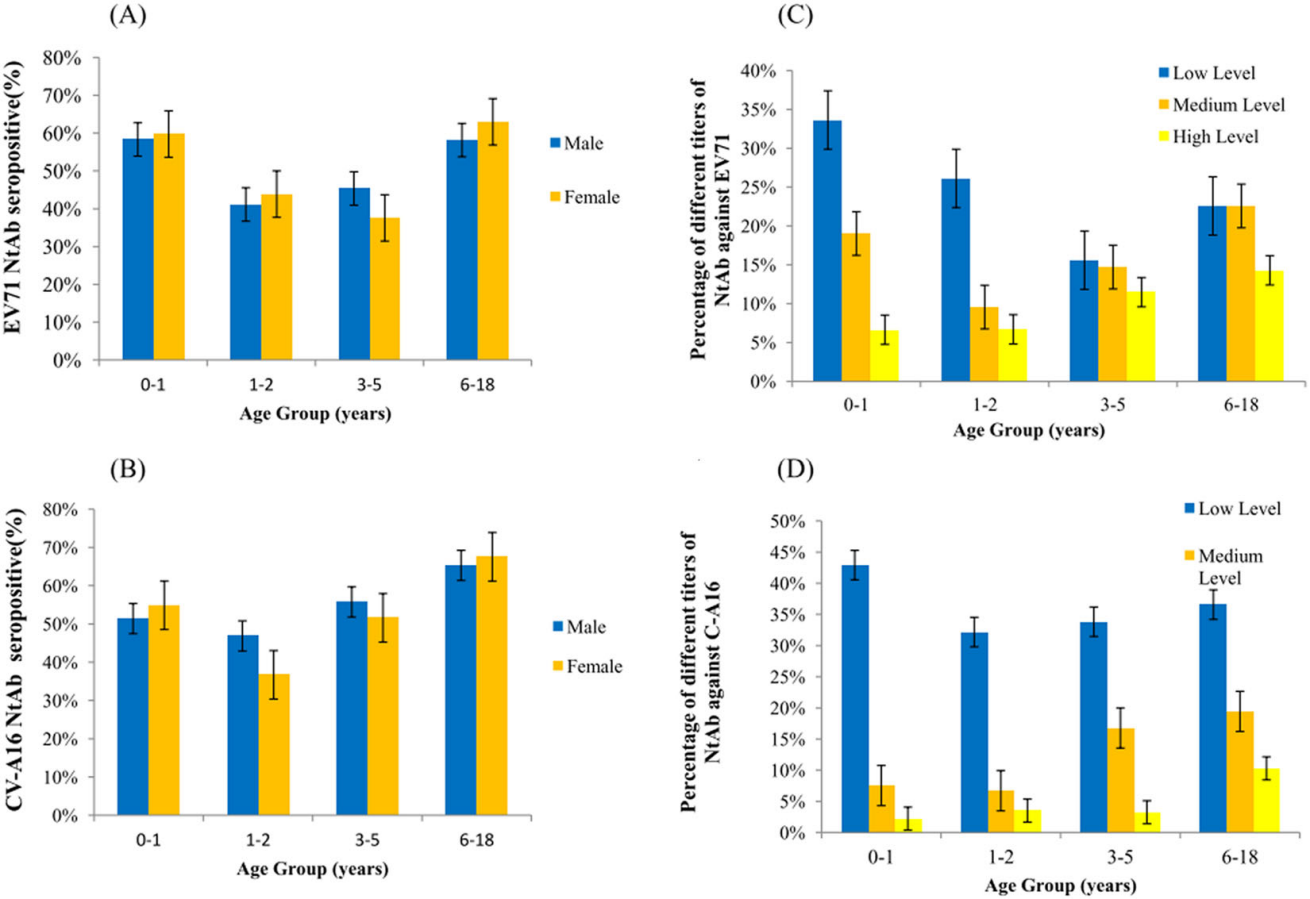
**Age-dependent seroprevalence and GMT values of EV71 and CV-A16 neutralising antibodies in healthy individuals in Shanghai, 2014–2016**. **a** Age-dependent seropositive rate of neutralizing antibody against EV71. **b** Age-dependent seropositive rate of neutralizing antibody against CV-A16. **c** Age-dependent GMT values of neutralizing antibody against EV71. **d** Age-dependent GMT values of neutralizing antibody against CV-A16.

Four different age groups were divided as 0–1 years, 1–2 years, 3–5 years and 6–18 years. Of these, 46.59% (410/880) and 49.43% (435/880) of the children aged <5 years had NtAbs to EV71 and CV-A16. No gender-specific differences were found in EV71 and CV-A16 seroprevalences. Boys aged 1–2 years had the lowest EV71 seroprevalence, and girls aged 3–5 years showed the lowest CV-A16 seroprevalence (Fig. 5a). The lowest CV-A16 seropositive rate was observed in the 1- to 2-year-old groups in both boys and girls (Fig. 5b). The seropositive rates of EV71 and CV-A16 NtAb titres were higher in the 6- to 18-year-old than in the younger groups, over 60% of whom had NtAbs.

Our results showed that the EV71 NtAb titre distributions among the different age groups was diverse (Fig. 5c). The proportions of high EV71 NtAb titres increased with age. A similar tendency was observed in the proportion of high CV-A16 NtAb titres. The 6- to 18-year-old possessed larger proportions of high EV71 and CV-A16 NtAb titres than the younger groups, and the 0- to 1-year-old had the largest proportion of low EV71 and CV-A16 NtAb titres, indicating a slow downward trend in transferred maternal antibody levels.

## Discussion

HFMD was classified as a C-class notifiable communicable disease in China in 2008. EV71 and CV-A16 became the dominant HFMD causative agents in Shanghai for some time after 2008^25,26^. Since the latter half of 2012, however, the positive rates of non-EV71 and non-CV-A16 EV-associated HFMD have grown rapidly, and these viruses became major factors in the Shanghai HFMD epidemic. Similar trends were also reported in Shanghai^28–30^ and other cities in China^12–18,31,32^. Recently, CV-A6 displaced EV71 and CV-A16 to become the predominant enteroviral serotype in Shanghai. Epidemiologists suggest two primary factors were involved in the regionally increased CV-A6 infection rates. First, after a long-running EV71/CV-A16 epidemic, the population may have accumulated high antibody titres against these serotypes; however, the antibody induced by the predominant serotypes, EV71 and CV-A16, cannot protect the susceptible population from the other serotypes^33^, which might impart selective pressure for other HEV serotypes to arise. Second, CV-A6 may have developed a highly recombinant strain that effectively sidesteps the human immune response. A novel recombinant CV-A6 of monophyletic lineage that was found during one outbreak of CV-A6-associated HFMDs in Shanghai led to a more generalised rash^30^. Chen et al.^34^ also reported changes to five unique nucleotides in the 3′-UTR, and 23 amino acids that were mainly located in the 3CD protein differed from the CA6 isolated in Taiwan between 2009 and 2010. More studies including whole genome sequencing of epidemic strains are warranted to determine whether variations affect the epidemic incidence of CV-A6. Long-term attention should be paid to preventing EV71 infections as they cause the most severe cases.

Seasonal circulation patterns among EV-predominant serotypes revealed that Shanghai, as with other southern cities in China, had semi-annual outbreaks of HFMD in May and September-October over the last few years^1,28,35^. Our data showed that EV71 and CV-A16, mainly circulating in May to July, were responsible for the summer HFMD epidemic. CV-A6 appears to have dominated the incidence peak in autumn and winter corresponding to the period from October to January. Some studies have also reported peak season variations between different years^7,36–39^. The current hypothesis explaining seasonal patterns includes host immune competence fluctuations mediated by seasonal factors, such as melatonin from vitamin D levels^40^, seasonal behavioural factors unrelated to weather, such as school attendance and indoor crowding^41^, temperature and relative humidity. However, factors such as school holidays or the beginning of a school term cannot account for the observed seasonal pattern. The incidence of EV71 infection was reported to correlate positively with higher temperatures and fair, sunny weather^1,42^.

Statistical analysis of the age and gender distribution in the present study showed that children under age five were the most susceptible to HFMD, which is consistent with previous reports^19,28,43,44^. Male predominance of HFMD was observed both in severe and mild cases in our study, possibly because of higher activity and poorer hygiene^45,46^. In severe cases, EV71 was the predominant causative agent, whereas CV-A16 and other EV strains were more common in mild cases.

We explored the potential link between aetiological characteristics and seroprevalence as well as titre distribution of NtAbs against EV71 and CV-A16; however, no link was found, likely due to the sampling method. To facilitate sampling, serum samples from some districts were recruited from the physical examination centre of the primary sentinel hospital. Some studies reported that sera collected from physical examinations were likely to have lower estimates compared with those of random samples. Sera from those who reported a previous history of HFMD or fever were excluded from the study and were less likely to be infected by EV71 or CV-A16^47^. Our study is the first systematic investigation into the seroprevalence and titre distribution of NtAbs against EV71 and CV-A16 pre- and post-HFMD epidemics during the three consecutive recent years in Shanghai. We believe these results will help to guide immunisation programmes against HFMD over the next several years. Children aged 1–5 years were found to be the most susceptible population to HFMD. Among them, children aged 1–2 years showed the highest incidence of severe HFMD and lower NtAb titres against EV71. Consequently, this cohort should be prioritised in any EV71 vaccination programme. Our study also found that EV71 and CV-A16 infections are relatively common. During the 2014–2016 periods, over 50% of healthy individuals showed evidence of being exposed to EV71, CV-A16 or both. Over one-third of individuals in this category were shown to have been exposed to both viruses. These populations may be an important potential source of infection as asymptomatic carriers.

Serum specimens collected during the pre-HFMD epidemic periods had lower EV71 and CV-A16 NtAb seropositive rates than those collected after the epidemics. These findings indicate that the enteroviral infection incidence is likely seasonal, consistent with our epidemiological findings. In accordance with the divergent trend of the major HFMD serotype, the overall EV71 seropositive rate exhibited a fluctuating downward trend, which also prompted the higher risk of EV71 infection over the next several years. High seroprevalence and percentages of high-level CV-A16 NtAbs confirmed a documented CV-A16 ‘silent’ epidemic in the 2015 post-HFMD epidemic period.

Our study revealed that infants younger than 1-year-old had higher NtAb seroprevalences against EV71 and CV-A16 than preschool children. This result is inconsistent with a study conducted in Shandong^27^ but is consistent with a seroepidemiology survey conducted in Shanghai in 2011^48^. High neonatal seroprevalence in Shanghai suggests higher maternal antibody levels against EV71 and CV-A16 infections, resulting from higher historical incidences of adult infections^49–51^. However, 6- to 18-year-old exhibited higher NtAb-positive rates against EV71 and CV-A16 than the younger groups, a finding consistent with previous studies^52,53^. Lower EV71 and CV-A16 seroprevalence among children aged 1–5 indicates that children in this age group are the most susceptible to EV71 and CV-A16 infections. The age pattern of seroprevalence was consistent with the age distribution in laboratory surveillance HFMD cases in our study.

Although the relationship between antibody titres and protection against reinfection remains unknown, high NtAb levels are always considered to indicate recent infection. In our study, the highest percentage of high-level EV71 NtAbs was in the 3- to 5-year-old group. Studies in other regions revealed similar trends, suggesting that the mean EV71 antibody titre in preschool children wanes after peaking at ~5 years of age^54,55^. In contrast to EV71, high CV-A16 NtAb levels were rarely observed in all age groups, and the highest percentage of high-level CV-A16 NtAb was in the 6- to 18-year-old group. Further study is required to determine whether immunity to EV71 and CV-A16 infection is reciprocally cross-protective.

This study retrospectively investigated the epidemiology and aetiology of HFMD in Shanghai over the past 5 years and showed EV71 and CV-A16 seroprevalence in healthy individuals in Shanghai. Several other studies demonstrated higher seroprevalence samples for enterovirus NtAbs among healthy individuals during major epidemic periods in Singapore, Cambodia, Japan and mainland China^47,52,56^. Similar trends were also observed in our study. Whether the seroprevalence of NtAb can be used to reliably predict the incidence of enterovirus outbreaks in the future needs further investigation. Although EV71 and CV-A16 are widely believed to be the most prevalent circulating serotypes in China, other serotypes such as CV-A6 and CV-A10 have recently surfaced in China and have become prominent serotypes leading to HFMD in some regions. Thus, monitoring long-term seroprevalence responses to enterovirus strains such as CV-A6 and CV-A10 should be emphasised in the future, as the EV71 vaccine has been successfully applied in Shanghai and other regions in China. We believe that it will help in understanding disease susceptibility and HFMD antibody titres among different age groups and provide a preliminary guide for future HFMD immunisation programmes.

## Electronic supplementary material

Table S1
