# Supplementary material for: Epidemiological and serological surveillance of hand-foot-and-mouth disease in Shanghai, China, 2012–2016
Source: Emerg Microbes Infect. 2018 Jan 24;7:8. doi: 10.1038/s41426-017-0011-z (PMC5837173; doi:10.1038/s41426-017-0011-z)
Supplement: Supplementary file 1 — Table S1 [file 41426_2017_11_MOESM1_ESM.pdf]

Table S1 PCR and sequencing primers used in this study.

Primer designation	Genomic region	Nucleotide sequence(5'-3')	Reference
EV71-VP1-F	EV71 VP1 gene	GCAGCCCAAAAGAACTTCAC	21
EV71-VP1-R	EV71 VP1 gene	AAGTCGCGAGAGCTGTCTTC	21
CV-A16-VP1-F	CV-A16 VP1 gene	ATTGGTGCTCCCACTACAGC	22
CV-A16-VP1-R	CV-A16 VP1 gene	GCTGTCCTCCCACACAAGAT	22
CV-A6-VP1-F	CV-A6 VP1 gene	CTTCGTAGTGCCACCAGATA	14
CV-A6-VP1-R	CV-A6 VP1 gene	GTGGCGAGATGTCGGTTTA	14
Sense 224	PanEV-VP1 Outer-primer	GCIATGYTIGGIACICART	23
Antisense 222	PanEV-VP1 Outer-primer	CICCIGGIGGIAYRWACAT	23
Sense AN89	PanEV-VP1 Inner-primer	CCAGCACTGACAGCAGYNGARAYNGG	23
Antisense AN88	PanEV-VP1 Inner-primer	TACTGGACCACCTGGNGGNAYRWACAT	23
486	Partial CV-A10 VP1gene	TGGTAICARACIAAITWYGTIGTNCC	24
488	Partial CV-A10 VP1gene	GTIGGRTAICCITCITARAACCAYTG	24
